# Supplementary material for: Berberine potentiates liver inflammation and fibrosis in the PI*Z hAAT transgenic murine model
Source: PLoS One. 2024 Sep 19;19(9):e0310524. doi: 10.1371/journal.pone.0310524 (PMC11412680; doi:10.1371/journal.pone.0310524)
Supplement: S1 Table — (DOCX) [file pone.0310524.s001.docx]

| **S1. Table: List of antibodies used for western blotting analysis.** | | |
| --- | --- | --- |
|  |  |  |
| Description | Supplier* | Cat# |
| GAPDH Antibody | PTG | 10494-1-AP |
| hAAT Rabbit Polyclonal Antibody | DAKO | A0012 |
| mTOR (7C10) Rabbit mAb | CST | 2983 |
| Phospho-mTOR (Ser2448) (D9C2) XP® Rabbit mAb | CST | 5536 |
| PERK (C33E10) Rabbit mAb | CST | 3192 |
| Anti-Phospho-PERK(T982) Rabbit antibody | LB | LS-C38147 |
| IRE1α (14C10) Rabbit mAb | NB | NB100-2323 |
| Anti-Phospho- IRE1α (S724) Rabbit antibody | MS | SAB5700519 |
| Caspase-3 Antibody | CST | 9662 |
| Caspase-8 Antibody | CST | 4790 |
| SQSTM1/p62 (D1Q5S) Rabbit mAb | CST | 39749 |
| LC3A/B Rabbit Antibody | CST | 12741 |
| * CST: Cell Signaling Technology (Danvers, MA, USA).  DAKO: DAKO (Bath, UK).  LB: Lifespan Biosciences (Seattle, WA, USA).  MS: MilliporeSigma (Burlington, MA, USA).  NB: Novus Biologicals |  |  |
| PTG: Proteintech Group (Rosemont, IL, USA). |  |  |
