# Supplementary material for: Berberine potentiates liver inflammation and fibrosis in the PI*Z hAAT transgenic murine model
Source: PLoS One. 2024 Sep 19;19(9):e0310524. doi: 10.1371/journal.pone.0310524 (PMC11412680; doi:10.1371/journal.pone.0310524)
Supplement: S2 File — (DOCX) [file pone.0310524.s003.docx]

**S3. Supporting information for Figure3. C**


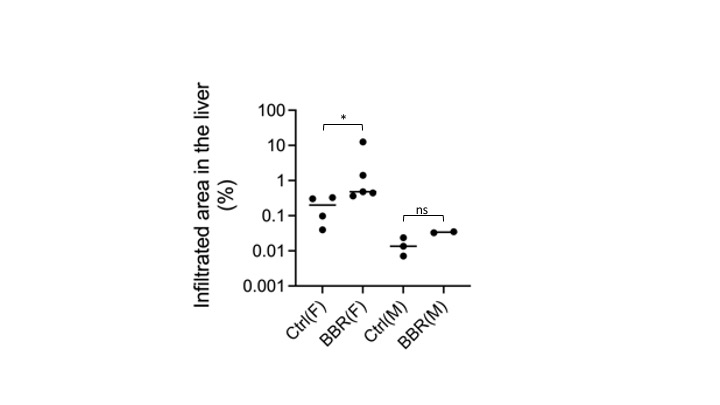


The quantification of the percentage of infiltrated area in the BBR-treated and

the untreated mice from the first *in vivo* experiment. The data from male and female mice were calculated separately because very rare inflammations were observed in male mice in both BBR-treated and untreated mice. Significant increases in percentage of inflammatory areas in the liver were observed in BBR-treated female mice (p = 0.016) but not in the male mice, likely because the low sample size in male mice.
