# Supplementary material for: Berberine potentiates liver inflammation and fibrosis in the PI*Z hAAT transgenic murine model
Source: PLoS One. 2024 Sep 19;19(9):e0310524. doi: 10.1371/journal.pone.0310524 (PMC11412680; doi:10.1371/journal.pone.0310524)
Supplement: S3 File — (DOCX) [file pone.0310524.s004.docx]

**S4. Supporting information for Figure5.**


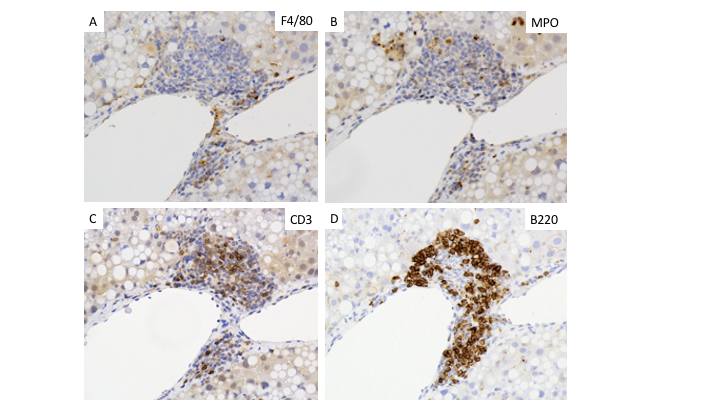


The inflammatory infiltrate was composed of predominantly lymphocytes.

Pi*Z transgenic mice were treated with 50 mg/kg/day of berberine chloride (n = 7,

5 females, 2 males) for 30 days. This figure presents the same immunohistochemical staining sections as presented in Figure 5 but were from another BBR-treated Pi*Z mouse liver.
