# Supplementary material for: Berberine potentiates liver inflammation and fibrosis in the PI*Z hAAT transgenic murine model
Source: PLoS One. 2024 Sep 19;19(9):e0310524. doi: 10.1371/journal.pone.0310524 (PMC11412680; doi:10.1371/journal.pone.0310524)
Supplement: S4 File — (DOCX) [file pone.0310524.s005.docx]

**S5. Supporting information for Figure 8**.


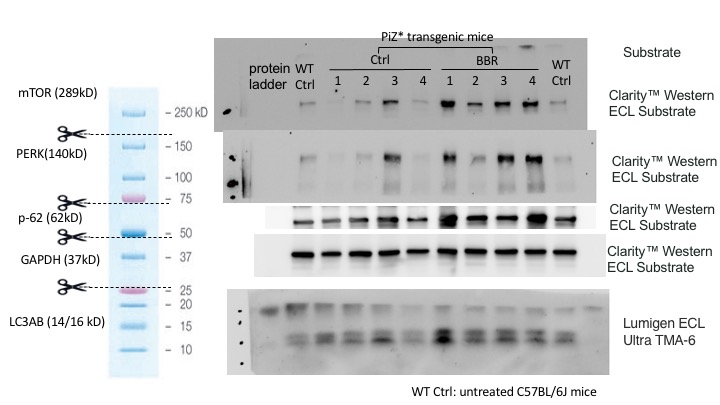


A


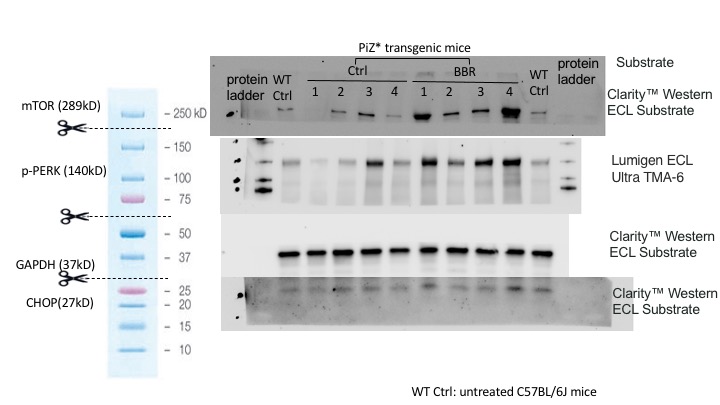


B


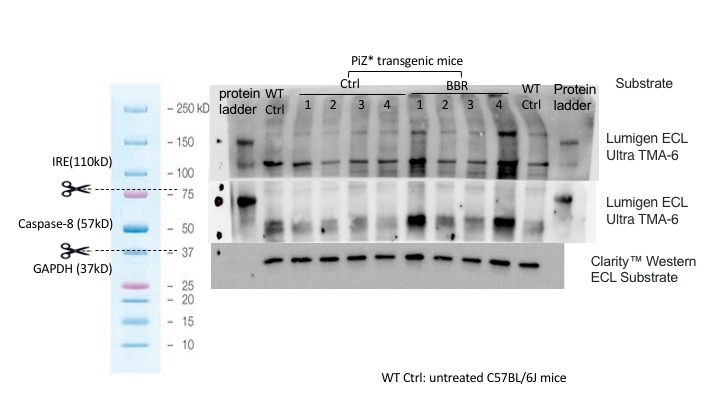


C

D


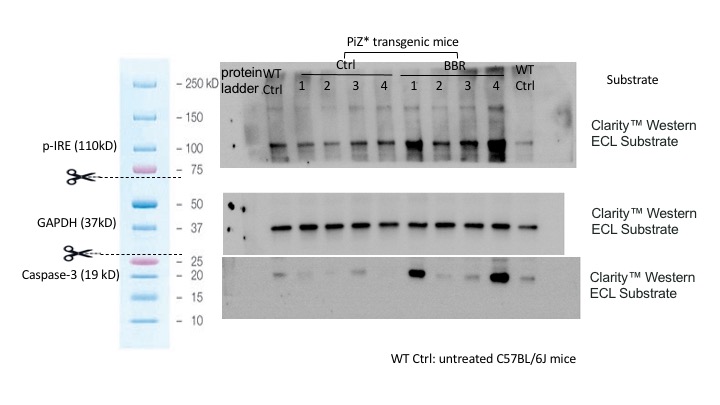


# Original uncropped scans of representative western blots displayed in Figure 5.
