# Supplementary material for: Berberine potentiates liver inflammation and fibrosis in the PI*Z hAAT transgenic murine model
Source: PLoS One. 2024 Sep 19;19(9):e0310524. doi: 10.1371/journal.pone.0310524 (PMC11412680; doi:10.1371/journal.pone.0310524)
Supplement: Attachment — Submitted filename: PONE-D-24-12616 response 7-23-24.docx [file pone.0310524.s007.docx]

PONE-D-24-12616

Berberine potentiates liver inflammation and fibrosis in the PI*Z hAAT transgenic murine model

PLOS ONE

Dear Professor Pullen,

Below is a point by point response to the reviewers comments. We appreciate the opportunity to resubmit our manuscript and believe the reviewers’ and the Editor’s suggestions have allowed us to improve the manuscript. Reponses are in **BOLD**.

- Are the images in figure 5 consecutive sections from the same liver? Is that truly representative if so?

**Yes. In Figure 5 the sections are from the same liver, and they are representative. To support this, we present the same immunohistochemical staining images as Figure 5 but from another BBR-treated Pi*Z mouse on S4. We believe that the macrophages play a critical role in initiation of inflammation and are then replaced by lymphocytes when we harvest livers.**

- There should be more said about the chosen *in vivo* dose either in the methods, discussion, or both. This should be in the context of other studies, what people typically would consume as a supplement, and also to the fact that BBR does not have good oral bioavailability. REF 33 somewhat gets at this as you already mentioned, and I understand that oral is the appropriate route (since that is how people consume it), but these are all factors that are important to consider for translational context.

**We totally agree with you. We updated the method and discussion sections accordingly.**

- The biochemical/mechanistic literature on glucose metabolism is probably the richest set of detailed cell biology information available for BBR. I was surprised not to see this synthesized in the context of a liver-specific study, particularly where a major sensor of cellular metabolism is discussed.

**Yes. The beneficial effects of berberine on blood glucose and lipid profile have been confirmed by many *in vivo* studies in diabetic and metabolic syndrome animal models. We measured blood glucose levels at the end of the experiments, and there were no abnormal blood glucose levels observed. There was no difference in blood glucose levels between the BBR-treated and the control groups, at least at this age of the Pi*Z mice. The oil-red O scores in the liver of BBR-treated Pi*Z mice were slightly low compared with the untreated group but statistically there was no significant difference between the two groups.**

1. Please ensure that your manuscript meets PLOS ONE's style requirements, including those for file naming. The PLOS ONE style templates can be found at
   <https://journals.plos.org/plosone/s/file?id=wjVg/PLOSOne_formatting_sample_main_body.pdf> and 
   <https://journals.plos.org/plosone/s/file?id=ba62/PLOSOne_formatting_sample_title_authors_affiliations.pdf>

**Yes.**

2. To comply with PLOS ONE submissions requirements, in your Methods section, please provide additional information regarding the experiments involving animals and ensure you have included details on (1) methods of sacrifice, (2) methods of anesthesia and/or analgesia, and (3) efforts to alleviate suffering.

**Updated.**

3. Thank you for stating the following financial disclosure:
   "MB. The Alpha-1 Foundation Research Professorship #007319."

Please state what role the funders took in the study.  If the funders had no role, please state: "The funders had no role in study design, data collection and analysis, decision to publish, or preparation of the manuscript." 
If this statement is not correct you must amend it as needed. 
Please include this amended Role of Funder statement in your cover letter; we will change the online submission form on your behalf.

**We have stated this in the cover letter.**

4. PLOS ONE now requires that authors provide the original uncropped and unadjusted images underlying all blot or gel results reported in a submission’s figures or Supporting Information files. This policy and the journal’s other requirements for blot/gel reporting and figure preparation are described in detail at <https://journals.plos.org/plosone/s/figures#loc-blot-and-gel-reporting-requirements> and <https://journals.plos.org/plosone/s/figures#loc-preparing-figures-from-image-files>. When you submit your revised manuscript, please ensure that your figures adhere fully to these guidelines and provide the original underlying images for all blot or gel data reported in your submission. See the following link for instructions on providing the original image data: <https://journals.plos.org/plosone/s/figures#loc-original-images-for-blots-and-gels>.
     
   In your cover letter, please note whether your blot/gel image data are in Supporting Information or posted at a public data repository, provide the repository URL if relevant, and provide specific details as to which raw blot/gel images, if any, are not available. Email us at plosone@plos.org if you have any questions.

**We included the original uncropped and unadjusted blot images in the Supporting information.**

5. We note that you have referenced (unpublished manuscript) on page 25, which has currently not yet been accepted for publication. Please remove this from your References and amend this to state in the body of your manuscript: (ie “Bewick et al. [Unpublished]”) as detailed online in our guide for authors
   <http://journals.plos.org/plosone/s/submission-guidelines#loc-reference-style>

**Actually, in the last paragraph on page 25 we cited two of our previous studies. The one we listed in the references is regarding the UPR, and it has been published. Another one is about the mTOR, that one has not been published and it is not included in the references. We updated the sentence in the main text, we hope it is more clear now.**

6. Please include captions for your Supporting Information files at the end of your manuscript, and update any in-text citations to match accordingly. Please see our Supporting Information guidelines for more information: <http://journals.plos.org/plosone/s/supporting-information>.

**Yes.**

Reviewers' comments:

Reviewer's Responses to Questions

**Comments to the Author**

1. Is the manuscript technically sound, and do the data support the conclusions?

The manuscript must describe a technically sound piece of scientific research with data that supports the conclusions. Experiments must have been conducted rigorously, with appropriate controls, replication, and sample sizes. The conclusions must be drawn appropriately based on the data presented.

Reviewer #1: Yes

Reviewer #2: Yes

2. Has the statistical analysis been performed appropriately and rigorously?

Reviewer #1: Yes

Reviewer #2: Yes

3. Have the authors made all data underlying the findings in their manuscript fully available?

The [PLOS Data policy](https://nam10.safelinks.protection.outlook.com/?url=http%3A%2F%2Fwww.plosone.org%2Fstatic%2Fpolicies.action%23sharing&data=05%7C02%7CYuanqing.Lu%40neurosurgery.ufl.edu%7C39faac7417e44ef7dfb208dc75f03330%7C0d4da0f84a314d76ace60a62331e1b84%7C0%7C0%7C638514921711769121%7CUnknown%7CTWFpbGZsb3d8eyJWIjoiMC4wLjAwMDAiLCJQIjoiV2luMzIiLCJBTiI6Ik1haWwiLCJXVCI6Mn0%3D%7C0%7C%7C%7C&sdata=oBTFQk12780TY1BuSKohKSlDlwpIkrgSjjeDFKG3I2s%3D&reserved=0) requires authors to make all data underlying the findings described in their manuscript fully available without restriction, with rare exception (please refer to the Data Availability Statement in the manuscript PDF file). The data should be provided as part of the manuscript or its supporting information, or deposited to a public repository. For example, in addition to summary statistics, the data points behind means, medians and variance measures should be available. If there are restrictions on publicly sharing data—e.g. participant privacy or use of data from a third party—those must be specified.

Reviewer #1: Yes

Reviewer #2: Yes

4. Is the manuscript presented in an intelligible fashion and written in standard English?

PLOS ONE does not copyedit accepted manuscripts, so the language in submitted articles must be clear, correct, and unambiguous. Any typographical or grammatical errors should be corrected at revision, so please note any specific errors here.

Reviewer #1: Yes

Reviewer #2: Yes

5. Review Comments to the Author

Please use the space provided to explain your answers to the questions above. You may also include additional comments for the author, including concerns about dual publication, research ethics, or publication ethics. (Please upload your review as an attachment if it exceeds 20,000 characters)

Reviewer #1: Lu et al. investigated the therapeutic effects and toxicities of berberine, a commonly used nature dietary supplement, on alpha-1 antitrypsin deficiency, using transgenic Pi*Z hepatocytes and Pi*Z mice. It a very interesting approach since there is evidence that berberine has both negative and positive effects on the liver. In my point of view, the study adds new and important information to the role of berberine on diseased individuals. There are some points to be clarified:
1) A major limitation is the low number of animals used in the study (n=7 including males and females) which is aggravated by the fact that there is a high level of variation in the BBR treated group and also that the observed effects appear to be gender dependent (see fig 3). This issue could be resolved by increasing the number of animals in each group.

**Thanks for spotting this. You are absolutely right. Unfortunately, our lab does not carry this strain of transgenic mice currently because of a transition of the laboratory and significant personnel changes. To resolve this problem, we combined the data from the Pi*Z mice in the first *in vivo* experiment (control group: 4 females, 3 males; BBR treatment group: 5 females, 2 males) with the data that were generated from the Pi*Z mice in the second *in vivo* experiment (control group: 2 females, 3 males; BBR treatment group: 6 females, 3 males) together and plotted a new graph as shown in Figure 3.C. We moved the old graph to the supplemental material as S3. We hope this will resolve the animal number issue. The new graph indicated that the inflammatory areas in the liver were significantly increased in both female and male mice after BBR treatment.**

2) It is well known that altered mitochondrial physiology and decreased ATP availability has been associated with some properties of berberine (antitumoral, antihyperglycemic and maybe toxic effects). Do authors think that the decrease in ATP content could also be related with the toxic effects observed in this study?

**We detected mitochondrial dysfunction in both Huh7.5Z cells and Pi*Z transgenic mouse livers, but the data will be used for another manuscript. We noticed the pharmacological effects of BBR targeting the mitochondria (Xiayi Fang, et al. Front Endocrinol (Lausanne). 2022; 13:982145). It could be important, but we did not investigate this pathway in this study because it is hard to explain the different responses to BBR between cells and animal model by this effect.**

3) Fig 2 – resolution was low, difficult to see the red points.

**It has been changed. Thank you.**

4) Fig 7 and 9 – The white arrows used are confusing with the histological image. Perhaps using black arrows would be better.

**Done, thanks.**

Reviewer #2: Alpha1-antitrypsin deficiency (AATD) is an inherited disorder arising due to mutations in alpha1-antitrypsin (AAT) gene leading to its polymerization and consecutive proteotoxic liver injury. Since there are no approved treatments for AATD-associated liver disease, Lu et al. tested the potential usefulness of berberine, a compound widely used in alternative medicine. This is a nice piece of work; however, a couple of issues need to be addressed prior to publication. In particular:
- The authors used RIPA buffer to measure intracellular AAT levels. Given that polymerized AAT is highly insoluble, it would be useful to check, whether RIPA buffer solubilizes as much AAT as buffers with higher SDS amounts. If not, this fact should be mentioned as a limitation.

**Very good point. 2% SDS has been used to extract insoluble Pi*Z AAT(** [**K S Graham**](https://pubmed.ncbi.nlm.nih.gov/?sort=date&term=Graham+KS&cauthor_id=2122976) [**1**](https://pubmed.ncbi.nlm.nih.gov/2122976/#full-view-affiliation-1)**,** [**A Le**](https://pubmed.ncbi.nlm.nih.gov/?sort=date&term=Le+A&cauthor_id=2122976)**,** [**R N Sifers**](https://pubmed.ncbi.nlm.nih.gov/?sort=date&term=Sifers+RN&cauthor_id=2122976)**. J Biol Chem. 1990 Nov 25;265(33):20463-8). Another method is to use 1% Triton X-100 and sonication to solubilize insoluble AAT polymers(** [**Jae-Koo An**](https://pubmed.ncbi.nlm.nih.gov/?sort=date&term=An+JK&cauthor_id=15619240)**,** [**Jeffrey H Teckman**](https://pubmed.ncbi.nlm.nih.gov/?sort=date&term=Teckman+JH&cauthor_id=15619240)**. Hepatology. 2005 Jan;41(1):160-7). RIPA buffer contains only 0.1% SDS, but it consists of 1% Triton X-100. We thought RIPA buffer and sonication is perhaps able to solubilize the insoluble AAT polymers.**

- Line 303-the statement that reduction of intracellular AAT levels was due to enhancing of the autophagy is not supported by data and should be tuned down. For example, one could say that it might be due to increased degradation.

**Thank you for the suggestion, we revised the statement**.

- It would be useful to report serum liver enzyme levels as a surrogate of hepatocellular injury.

**Yes. We measured serum ALT levels at the endpoint of the experiments by using an ALT ELISA kit, but we failed to detect the elevation of ALT in the blood of BBR-treated mice. We couldn’t figure out the possible causes. It could be due to the kit sensitivity or we missed the peak of the ALT curve.**

- It would be useful to use a second method of fibrosis quantification/stellate cell activation (i.e., collagen RT-PCR, hydroxyproline assay or a comparable technique).

**Thank you for your suggestion. We agree with your point, but it is difficult to conduct a new experiment on this project due to the significant personnel changes in the laboratory that both first author and corresponding author were involved in.** **We hope you understand this situation.**
- In addition to PAS-D staining, it would be nice to quantify the intrahepatic AAT levels in treated/non-treated animals via immunoblotting.

**We tried to quantify the AAT and 2C1 IHC images but due to the high density of the globules that appeared in some Pi*Z mice, which are also stained by the AAT and 2C1 antibodies, this made it very difficult to eliminate the globules from the stained cells.**

6. PLOS authors have the option to publish the peer review history of their article ([what does this mean?](https://nam10.safelinks.protection.outlook.com/?url=https%3A%2F%2Fjournals.plos.org%2Fplosone%2Fs%2Feditorial-and-peer-review-process%23loc-peer-review-history&data=05%7C02%7CYuanqing.Lu%40neurosurgery.ufl.edu%7C39faac7417e44ef7dfb208dc75f03330%7C0d4da0f84a314d76ace60a62331e1b84%7C0%7C0%7C638514921711779203%7CUnknown%7CTWFpbGZsb3d8eyJWIjoiMC4wLjAwMDAiLCJQIjoiV2luMzIiLCJBTiI6Ik1haWwiLCJXVCI6Mn0%3D%7C0%7C%7C%7C&sdata=6tka2ep5kZLbBak4OcFKytX5H3iASc%2FlojxM8bC7szU%3D&reserved=0)). If published, this will include your full peer review and any attached files.

If you choose “no”, your identity will remain anonymous but your review may still be made public.

Do you want your identity to be public for this peer review? For information about this choice, including consent withdrawal, please see our [Privacy Policy](https://nam10.safelinks.protection.outlook.com/?url=https%3A%2F%2Fwww.plos.org%2Fprivacy-policy&data=05%7C02%7CYuanqing.Lu%40neurosurgery.ufl.edu%7C39faac7417e44ef7dfb208dc75f03330%7C0d4da0f84a314d76ace60a62331e1b84%7C0%7C0%7C638514921711788371%7CUnknown%7CTWFpbGZsb3d8eyJWIjoiMC4wLjAwMDAiLCJQIjoiV2luMzIiLCJBTiI6Ik1haWwiLCJXVCI6Mn0%3D%7C0%7C%7C%7C&sdata=al7t6tT0Bw8Swf3RcV8uKyDQTfYAQ1isMWobEnawaS0%3D&reserved=0).

Reviewer #1: No

Reviewer #2: No

Sincerely,

Mark Brantly, MD

Professor of Medicine, Molecular Genetics and Microbiology

Alpha One Foundation Research Professor

University of Florida College of Medicine

1600 SW Archer Rd Rm M454A

Gainesville, FL 32610
